# Increased biological antioxidant potential in the cerebrospinal fluid of transient global amnesia patients

**DOI:** 10.1038/s41598-021-95343-6

**Published:** 2021-08-05

**Authors:** Takayuki Kawai, Ryuji Sakakibara, Yosuke Aiba, Fuyuki Tateno, Tsuyoshi Ogata, Setsu Sawai

**Affiliations:** 1 Research Advancement Unit, Sakura Medical Center, Toho University, Sakura, Japan; 2 Neurology, Internal Medicine, Sakura Medical Center, Toho University, 564-1 Shimoshizu, Sakura, 285-8741 Japan

**Keywords:** Biochemistry, Neuroscience, Neurology

## Abstract

Oxidative stress may accompany the pathological process in transient global amnesia (TGA). We measured the biological antioxidant potential (BAP) in the cerebrospinal fluid (CSF) of TGA patients. We enrolled 13 TGA patients (7 men, 6 women; mean age 65.0 years [48–70 years]) and 24 control subjects (12 men, 12 women; mean age 38.2 years [17–65 years]; age did not correlate with csfBAP in this group). We performed brain MRI in all TGA patients, and CA1 lesions were noted by MRI in 5 subjects. We measured csfBAP, total antioxidant properties, in all TGA patients and controls. csfBAP levels were higher in TGA patients than in controls (p = 0.024, 0.028). csfBAP levels in TGA patients did not differ between MRI-positive and -negative subgroups. Elevated csfBAP levels were observed in TGA patients, suggesting that oxidative stress may have a role in the pathogenesis of TGA.

## Introduction

Transient global amnesia (TGA)^1^ is defined by a sudden onset of an anterograde and retrograde amnesia that lasts up to 24 h^2, 3^. Since then, several aetiological factors, such as migraine-related mechanism (the spreading depression), have been suggested to be causative factors. Other proposed factors include epileptic cause (transient epileptic amnesia), focal ischaemia, venous flow abnormalities, and genetic factors (Dandapat et al.^4^, 2 sister cases, gene location not mentioned; Arena et al.^5^, familial cases review, gene location not mentioned, Pradott et al.^6^, a case, later developing full clinical manifestation of CADASIL with positive Notch3 gene). More recently, the CA1 region of the hippocampus was shown to have abnormal signal intensity in up to 50% of patients within a 1-week window after onset^2, 3^, suggesting that metabolic derangement occurs in the brain, although the precise mechanism remains unclear. Oxidative stress has attracted attention in neurologic diseases such as encephalitis, stroke, neurodegenerative disease, etc^7–9^. It is therefore postulated that oxidative stress may also accompany the pathological process in TGA. However, no literature exploring this possibility has been available so far. To explore this issue, we measured biological antioxidant potential (BAP) in the cerebrospinal fluid (CSF) of TGA patients and examined the relationship between csfBAP levels and neuroimaging abnormalities in the hippocampal CA1 regions of TGA patients and controls.

## Materials and methods

This is a retrospective study at a university clinic, and all patients were referred patients. CSF sampling had been administered to rule out other causes (i.e., infectious). For inclusion in the study, patients had to have all of the following: (1) diagnosis with TGA according to the published criteria^2, 3^, (2) a visit to our clinic within 1 or 2 days after the onset of TGA, together with CSF sampling, (3) a brain magnetic resonance imaging (MRI) scan within 1–7 days after onset, with at least two attempts to obtain a positive scan, in order to obtain positive scan, and (4) a standard neurological examination, cognitive tests including the Mini-Mental State Examination (MMSE; 0–30 scale, normal > 24), the Frontal Assessment Battery (FAB; 0–18 scale; normal > 16), and Alzheimer’s Disease Assessment Scale, cognitive subscale (ADAS-cog; 0–70 scale, normal < 10), blood test, and electroencephalography [data not shown]. Exclusion criteria were: (1) neurological diseases other than TGA, (2) a systemic infectious or inflammatory disease that might affect csfBAP, and (3) contraindications for a lumbar puncture, e.g., severe lumbar spondylosis, infection at the lumbar area, etc. This study was approved by the Ethics Committee in Sakura Medical Center, and was conducted according to the Declaration of Helsinki. Informed consent was obtained from all participants.

Our study included 13 TGA patients (7 men, 6 women; mean age 65.0 years [48–70 years]) and 24 control subjects (12 men, 14 women; mean age 38.2 years [17–65 years] during a 3-year period. Control subjects were those who had been referred to our hospital as having suspected meningitis; and in whom neurological examination and CSF findings were all normal. Three of control subjects were taking antihypertensive and/or anti-dyslipidemia drugs.

We performed brain MRI in all TGA patients and control subjects. The time window of MRI in TGA patients was within 7 days after the onset (the first scan: day 0, 2, day 1, 9, day 2, 2; the second scan if available, day 4–6, 3). MRIs were performed using a 3.0-T MRI scanner (MAGNETOM Skyra; Siemens AG, Erlangen, Germany) with a standard eight-channel head coil. The sequence were standard diffusion-weighted and corresponding apparent diffusion coefficient (ADC) map, T1-weighted, fluid-attenuated inversion recovery (FLAIR), and T2-weighted images in the axial, coronal and sagittal planes including the hippocampal/parahippocampal area. Diffusion-weighted images were acquired using a fat-saturated single-shot echo-planar imaging sequence along 30 and 60 motion-probing gradient directions with b values of 1000 and 2000 s/mm^2^, respectively.

We measured csfBAP (total antioxidant properties) in all TGA patients and control subjects. In order to get reliable data, we obtained CSF samples early in the morning to the extent possible without taking foods and medicine. CSF samples were collected by a lumbar puncture, which were frozen in polypropylene tubes and stored at − 80 °C until assay. BAP is the quantity of molecules with antioxidative potency measured by a Free Radical Elective Evaluator (FREE^R^; Wismer Co Ltd, Tokyo, Japan; equivalent to BAP, Diacron Ltd, Grosseto, Italy)^7–9^. BAP measurement is based on the assumption that CSF samples [CSF (e−)] contain electron-donating, antioxidant molecules that can bind to the added ferric chloride (FeCl_3_; a source of ferric ions, Fe^3+^), leading to detectable chromatic changes that are directly proportional to the ability of CSF to reduce reactive oxygen species (ROS). By photometrically assessing the intensity of decolonization, the amount of reduced ferric ions can be adequately evaluated, allowing effective measurement of reducing ability or antioxidant potential of tested CSF sample. The whole sequence is automated, and antioxidant activity levels can be evaluated fast and easily. Necessary CSF sample volume is 10 μl. After a short incubation of 5 min, such solutions decolorized and the intensity of this chromatic change is considered directly proportional to the ability of CSF during the incubation to reduce ferric ions to ferrous ions. The BAP test was performed on the following standard working conditions of optical spectroscopy probes: wavelength 505 (range 500–510) nm, optical path 1 cm, and temperature 37 °C. The results are expressed as micromol/l^7–9^. Non-parametric statistical analyses were performed by the Mann–Whitney U test. The level of statistical significance was p < 0.05.

## Results

**Figure 1:**
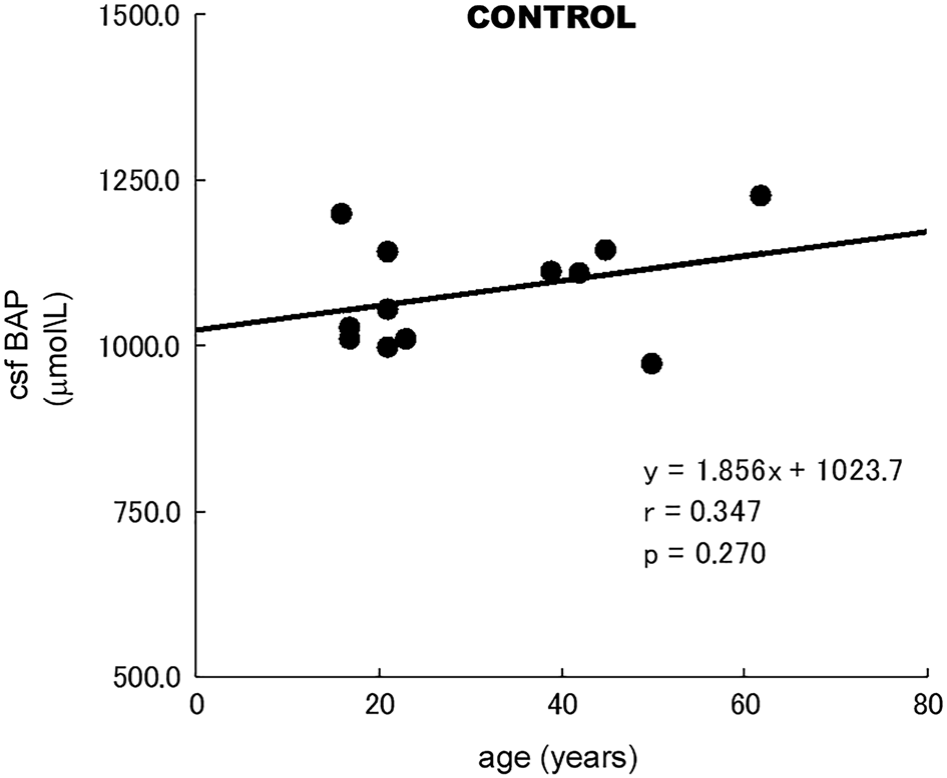
Relationship between csfBAP and age in the control group. *csf* cerebrospinal fluid, *BAP* biological antioxidant potential. There was no relationship between csfBAP and age in the control group.

Since the age of control was younger than that of TGA patients, we checked the effect of age on CSF BAP levels. CSF BAP level was not related with age in the control group (Fig. 1).

On arrival to our clinic (after cessation of TGA episode), the cognitive test results of the patients were: mean MMSE value of 27 (range 23–29), mean FAB value of 16 (14–18), and mean ADAS-cog value of 4 (0–6). We did not perform cognitive tests in control subjects since they visited our clinic because of fever and headache. None of control subjects revealed apparent cognitive decline/consciousness abnormality.

**Figure 2:**
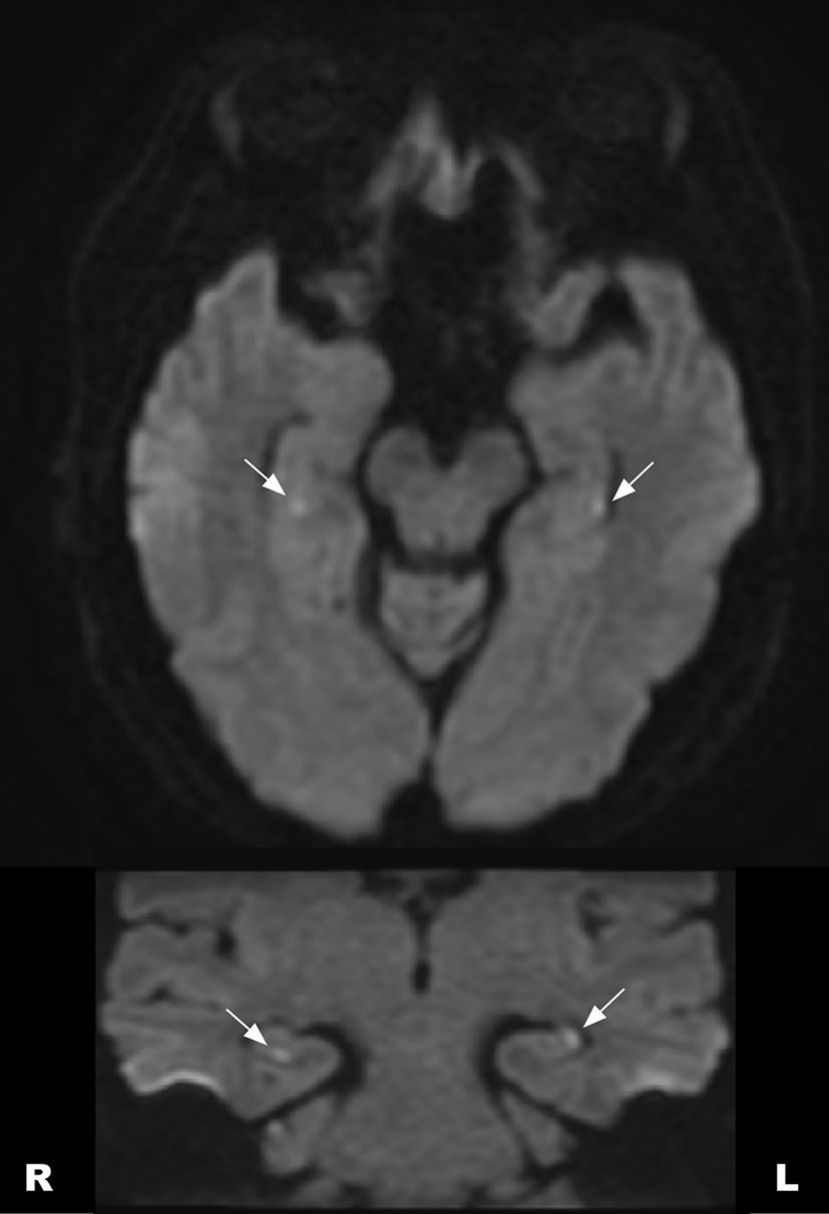
Representative diffusion-weighted MRI showing lesions in a TGA patient. Upper, axial slice; lower, coronal slice. *TGA* transient global amnesia, *MRI* magnetic resonance imaging. Arrows indicate hippocampal CA1 lesions.

Brain MRI showed CA1 lesions in 5 subjects by diffusion-weighted MRI, while the size of lesions was small (2–3 mm) (Fig. 2). The lesion laterality was right in 1, left in two, and bilateral in two. According to the relationship between diffusion positivity and time window of MRI acquisition in TGA patients, it was 1 in 2 (day 0), 3 in 9 (day 1), 0 in 2 (day 2) and 1 in 3 (day 4–6). Control subjects had normal MRI finding.

**Figure 3:**
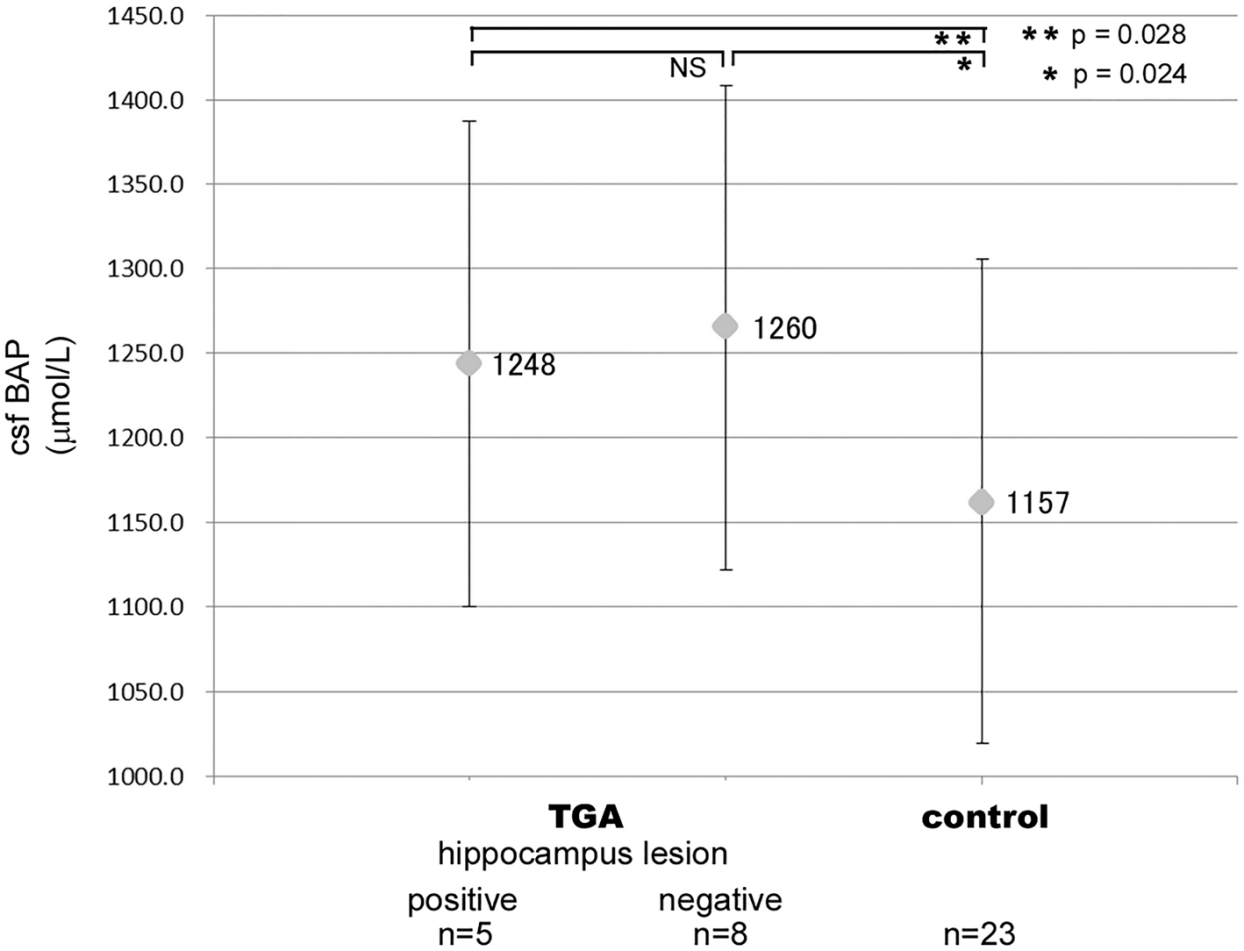
csf BAP in TGA patients and controls. *csf* cerebrospinal fluid, *BAP* biological antioxidant potential, *TGA* transient global amnesia, *Hippocampus lesion* hippocampus lesion by a diffusion-weighted MRI scan. Statistical analysis was made by the Mann–Whitney U test. csfBAP value in TGA (regardless of MRI hippocampus lesion) was higher than that in the control group (p = 0.024, 0.028). However, there was no difference in csfBAP values between hippocampus lesion-positive and -negative TGA patients.

The csfBAP values were higher in TGA patients than in controls (p = 0.024, 0.028) (Fig. 3). The csfBAP levels in TGA patients did not differ between hippocampal CA1 lesion-positive and -negative subgroups. The results of blood analysis, and electroencephalography were normal in all patients.

## Discussion

Biomarkers of oxidative stress are often short-lived and labile. Sensitive but chemically and metabolically stable markers are needed, particularly for CSF. To our knowledge, this is the first study to show increased csfBAP in TGA patients (p = 0.024, 0.028). The finding may support the idea that CSF oxidative stress changes directly relate with the pathological process in the TGA brain.

Thus far, several potential factors, such as specific genes^4–6^, migraine-related mechanisms, epileptic phenomena, and venous flow abnormalities have been identified^2, 3^. The hippocampal CA1 region may show abnormality on MRI scan^2, 3^, either unilaterally or bilaterally, suggesting metabolic derangement and/or focal ischaemia in the TGA brain. In reported cases of acute amnesia due to hippocampus stroke, of which the lesion size ranged from 10 mm to more than 50 mm^10^, it is postulated that lesion size > 10 mm might account for clinical amnestic syndrome. With respect to these findings, and the fact that half of TGA cases were MRI-negative, true lesion area seems to be larger than the area that MRI could visualize (2–3 mm, indicating ‘a radiographical footprint’). Parallel with these findings, it is reported that functional connectivity between the hippocampal CA1 region and other brain areas may change^11^. Bartsch et al. performed magnetic resonance spectroscopy (MRS) at the hippocampal CA1 region of seven TGA patients^12^. They found an increased lactate peak in 3 of 4 patients with hippocampal CA1 lesions, but in 0 of 3 patients without hippocampal CA1 lesions. Since lactate is a marker of anaerobic glycolysis, an increased lactate peak indicates acute metabolic stress in the CA1 neurons in TGA. To date, there is no animal model of TGA. However, MRS studies in experimental rat mild traumatic brain injury (TBI) showed aberrant bioenergetics, glutamate excitotoxicity, and increases in oxidative stress markers due to mitochondrial damage^13, 14^. In these mild TBI model animals, an elevated lactate peak by MRS was related with poor memory recovery^13^. Therefore, increased csfBAP in the present study supports the idea that CSF oxidative stress may have a pivotal role in the pathogenesis of TGA.

What is the effects of oxidative stress in TGA? Within the brain, it is well recognized that mitochondria have crucial roles in energy metabolism regulation, cell cycle, survival and death, apoptosis, generation of reactive oxygen species (ROS), and calcium homeostasis. Superoxide anion is the precursor of ROS such as hydrogen peroxide and hydroxyl radical, which can damage lipids, nucleic acids, and proteins, becoming critical players in the progression of neuro-degeneration, atherosclerosis and stroke. Among these, ischemic stroke is a disease in which ischemic/reperfusion injuries do occur, where a cerebral region is deprived of oxygen due to an obstruction of a blood vessel^15, 16^. Parallel with intra-arterial intervention and thrombolysis, newer mitochondria-targeted neuroprotective therapeutics, such as cationic arginine-rich peptides and edaravone (a free radical scavenger), are now being available for ischemic stroke^17–19^. Among these, edaravone is a new antioxidant and hydroxyl radical scavenger that has been used to treat amyotrophic lateral sclerosis (ALS). Although there is evidence that edaravone improves clinical outcomes of patients with acute ischaemic stroke, it is not yet widely accepted. A recent meta-analysis of seven randomized controlled trials including European studies with 2069 patients, edaravone showed positive impact on mortality (*p* < 0.01) and improvement of neurological impairment at 3 months (*p* < 0.01); and any treatment-related adverse events (not statistically significant)^20^. Therefore, if we postulate that TGA and brain infarction might share the same ischemia-induced pathology, it seems reasonable to assume that these drugs might shorten the duration of TGA and enhance patients’ quality of life in future.

The limitations of our study obviously include a small number of patients; and a lack of age-adjustment between control and TGA groups. Therefore the false positive cannot be excluded. However, csfBAP was not related with age (10–60 years) among the control group; therefore, the comparison between control and disease groups seems decent. Another limitation is that csfBAP in TGA in the present study did not differ between hippocampal CA1 lesion-positive and -negative subgroups. We did not know the exact reason for this. However, one explanation might be that the lesion is so small (2 to 3 mm). In order to explore this, further studies with a larger number of patients are needed. Also, the time window of MRI in TGA patients of our study was within 7 days after the onset. It we perform an MRI scan as early as possible, the detection rate might increase and bring about relationships. The limitations include the lack of cognitive assessment of controls. The limitations also include that we measured antioxidant activity BAP alone, without measuring oxidative stress. Previously, no direct oxidative stress measurement in CSF is available in TGA. However, looking at serum in other diseases, in post-surgery (acute phase), both serum d-ROM (oxidative stress) and BAP (antioxidant activity) increased^21^. Also, in MELAS (myopathy, encephalopathy, lactic acidosis and stroke-like episodes) (acute exacerbation phase), both serum d-ROM and BAP increased^22^. Thus, increased markers of oxidative stress and antioxidant activity might reflect on-going oxidative insult. As for the focal ischemia mechanism of TGA, the previous supportive data is scarce, and no conclusion can be obtained even though oxidative stress might have contributed to the occurrence of TGA. We still need more studies to explore the nature of TGA.

In conclusion, the results of the present study showed increased csfBAP in TGA, suggesting that oxidative stress may have a role in the pathogenesis of TGA.

## Data Availability

2011-058 by the Ethics Committee in Sakura Medical Center, Toho University.
